# Characterization of the influence of age on GABA_A_ and glutamatergic mediated functions in the dorsolateral prefrontal cortex using paired-pulse TMS-EEG

**DOI:** 10.18632/aging.101178

**Published:** 2017-02-16

**Authors:** Yoshihiro Noda, Reza Zomorrodi, Robin F. H. Cash, Mera S. Barr, Faranak Farzan, Tarek K. Rajji, Robert Chen, Zafiris J. Daskalakis, Daniel M. Blumberger

**Affiliations:** ^1^ Temerty Centre for Therapeutic Brain Intervention, Centre for Addiction and Mental Health, Toronto, Ontario, M6J 1H4, Canada; ^2^ Department of Psychiatry, University of Toronto, Toronto, Ontario, M5T 1R8, Canada; ^3^ Monash Alfred Psychiatry Research Centre, Monash University Central Clinical School and The Alfred, Melbourne 3004, Australia; ^4^ Division of Neurology, Department of Medicine, University of Toronto, Division of Brain, Imaging and Behaviour – Systems Neuroscience, Krembil Research Institute, University Health Network, Toronto, M5T 2S8, Ontario, Canada; ^5^ Campbell Family Mental Health Research Institute, Centre for Addiction and Mental Health, Toronto, Ontario, M5T 1R8, Canada

**Keywords:** aging effect, TMS–EEG, short interval intracortical inhibition (SICI), intracortical facilitation (ICF), dorsolateral prefrontal cortex (DLPFC)

## Abstract

Gamma-aminobutyric acid (GABA)ergic and glutamatergic neurotransmissions in the prefrontal cortex decreases with age. Further, cognitive function mediated through the dorsolateral prefrontal cortex (DLPFC) also declines with age. Although neuroimaging studies have demonstrated decreased levels of these substances, direct neurophysiological data investigating the effect of aging in the DLPFC in human subjects is lacking. The advent of transcranial magnetic stimulation (TMS) combined with electroencephalography (EEG) has allowed for the assessment of functional neurotransmission in vivo. In the present study, we examined short interval intracortical inhibition (SICI) and intracortical facilitation (ICF) in a group of older adults (> 60 yrs) to evaluate the strength of GABA_A_ and glutamate-mediated neurotransmission in the DLPFC, compared to younger adults (18-59 yrs). Older adults showed an increase of amplitude of N100 by the SICI paradigm, while N45 amplitude was increased and N100 amplitude was decreased by ICF. Moreover, these modulations significantly correlated with age. Our findings provide evidence for age-related alterations of excitatory and inhibitory functions in the prefrontal cortex in healthy adults. Future studies may aim to explore these neurophysiological relationships in the DLPFC in pathological forms of aging that affect cortical functioning such as mild cognitive impairment and Alzheimer's disease.

## INTRODUCTION

Neuroimaging studies of the human prefrontal cortex using magnetic resonance spectroscopy (MRS) [1, 2] have demonstrated that gamma-aminobutyric acid (GABA) and glutamate levels decrease with age. Specifically, neurotransmitter levels of GABA and glutamate in the dorsolateral prefrontal cortex (DLPFC) have been shown to be decreased in older adults compared to younger adults [2]. However, there are no studies that have investigated the effect of age on GABAergic and glutamatergic neurophysiological functions in the prefrontal cortex.

Paired-pulse transcranial magnetic stimulation (TMS) paradigms have been used to non-invasively explore intracortical inhibitory and facilitatory mechanisms originally from the primary motor cortex (M1). Short-interval intracortical inhibition (SICI) and intracortical facilitation (ICF) are two such paradigms to index GABA type A (GABA_A_) receptor-mediated inhibition and glutamate receptor-mediated excitation, respectively. In contrast to MRS studies [2], neurophysiological studies have reported mixed effects of normal aging on SICI [3-5]. For example, Peinemann and colleagues (2001) have reported an age-related decrease in SICI, two other studies observed no age-related effects [4, 6], and two studies observed an age-related increase in SICI [5, 7]. These mixed results may be attributed to age-related changes in cortical gene expression in the GABAergic system [8]. Specifically, the dysregulation of glutamic acid decarboxylase (GAD65) protein with age, which plays an important role in GABA synthesis, may induce an increased expression of GABA receptors as a self-regulated compensatory effect in the cortex for the reduced GABA release during normal aging [8]. In contrast, regarding age-related changes in ICF, one study has reported that older adults exhibit less ICF than younger adults [7]. This may be due to an age-related decrease of glutamate content, specifically the density of glutamatergic N-methyl-D-aspartate (NMDA) receptors, in the cerebral cortex, which is mainly thought to originate from changes in metabolic activity rather than glutamatergic neurotransmission with age [9]. In addition, very few studies have examined the neurophysiological relationship between glutamate receptor mediated excitation indexed with the ICF paradigm and GABA_A_ receptor-mediated inhibition indexed with the SICI paradigm of M1 in human subjects [10].

Recently, we have established a combined TMS–EEG technique to measure the SICI and ICF paradigms from the DLPFC [11]. The development of this technique allows for the assessment of GABAergic and glutamatergic functioning in a cortical region involved in the effects of aging on cognition and emotion.

In this study, therefore, we sought to explore the effects of aging on SICI and ICF from the DLPFC using combined TMS-EEG. We hypothesized that older adults would have reduced SICI and ICF compared to younger adults. We also explored the effect of age on overall balance of excitatory and inhibitory functioning in all participants.

## RESULTS

### Modulations of TMS-evoked potential (TEP) amplitude with SICI and ICF paradigm in older adults

#### DLPFC-SICI in older adults

**Figure 1:**
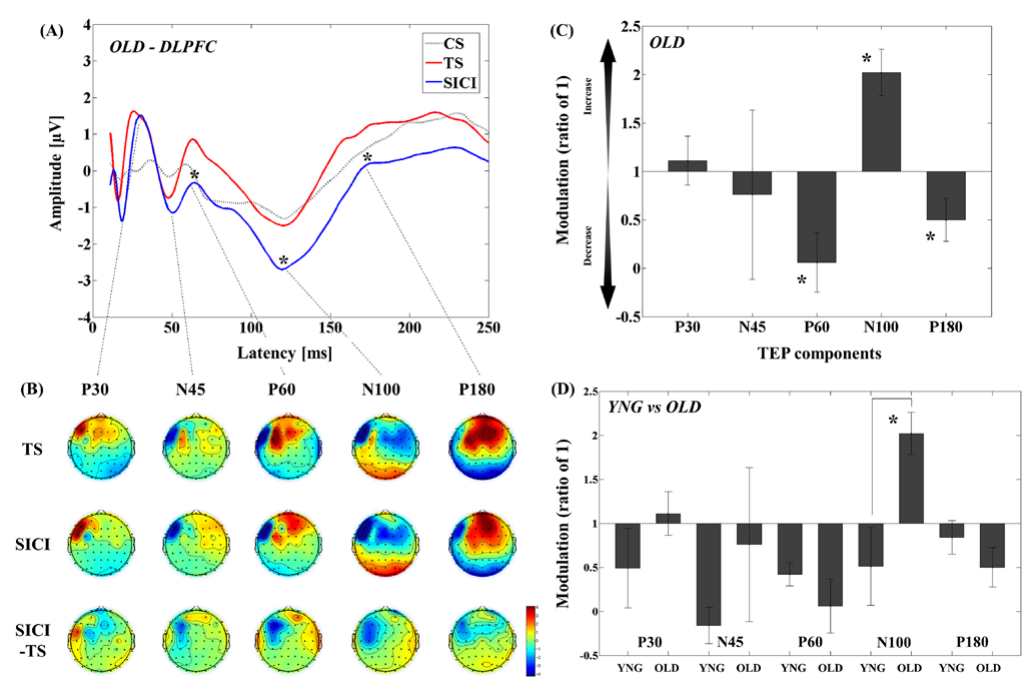
Modulation of TEPs by the DLPFC-SICI paradigm in older adults **(A)** The graph depicts TEP traces averaged across the older adults for subthreshold TMS (black dot line: CS), unconditioned TMS (red line: TS) and conditioned TMS (blue line: SICI; ISI = 2ms) at the left frontal ROI. **(B)** The illustration shows the EEG topographical plots for conditions of TS alone, SICI, and the difference between TS and SICI obtained from the DLPFC-SICI experiment. Each vertical column depicts the TEP topoplots for P30, N45, P60, N100, and P180 component from left to right. **(C)** The bar graph shows modulatory effects of the DLPFC-SICI on TEPs in the older adults. The ANOVA and post-hoc analyses revealed that there are significant modulations (p < 0.05) in P60, N100, and P180 TEPs with the DLPFC-SICI paradigm. **(D)** The bar graph showing cross-sectional comparisons between younger and older adults in the DLPFC–SICI paradigm. The older adults show a significant facilitation of amplitude of N100 TEP than the younger adults (p < 0.05).

The results of averaged TEP traces of older adults for subthreshold stimulus (conditioning stimulus (CS) alone; 80% of resting motor threshold (RMT)), suprathreshold stimulus (test stimulus (TS) alone; intensity to induce 1mV peak-to-peak motor evoked potential (MEP) amplitude), and conditioned stimulus (SICI: CS&TS) in SICI paradigm are shown in Figure 1A. A 3–way analyses of variance (ANOVA) and post-hoc analyses (α–level: 0.05) for TEP values of the SICI indicated significant differences between TS and CS&TS on P60 (t_11_ = 5.019, p < 0.0001; TS > SICI; Cohen's *d* = 1.19, Power (1-β) = 0.963), N100 (t_11_ = 4.394, p = 0.001; TS > SICI; Cohen's *d* = 1.54, Power (1-β) = 0.998), and P180 (t_11_ = 2.984, p = 0.012; TS > SICI; Cohen's *d* = 0.69, Power (1-β) = 0.587). TEPs at the left frontal region of interest (ROI) (Supplementary Table S1). Figure 1B shows the EEG topographical plots for conditions of TS, SICI, and the difference between TS and SICI in the SICI experiment. The TEP amplitude modulations at the left frontal ROI are demonstrated in Figure 1C.

#### DLPFC-ICF in older adults

**Figure 2:**
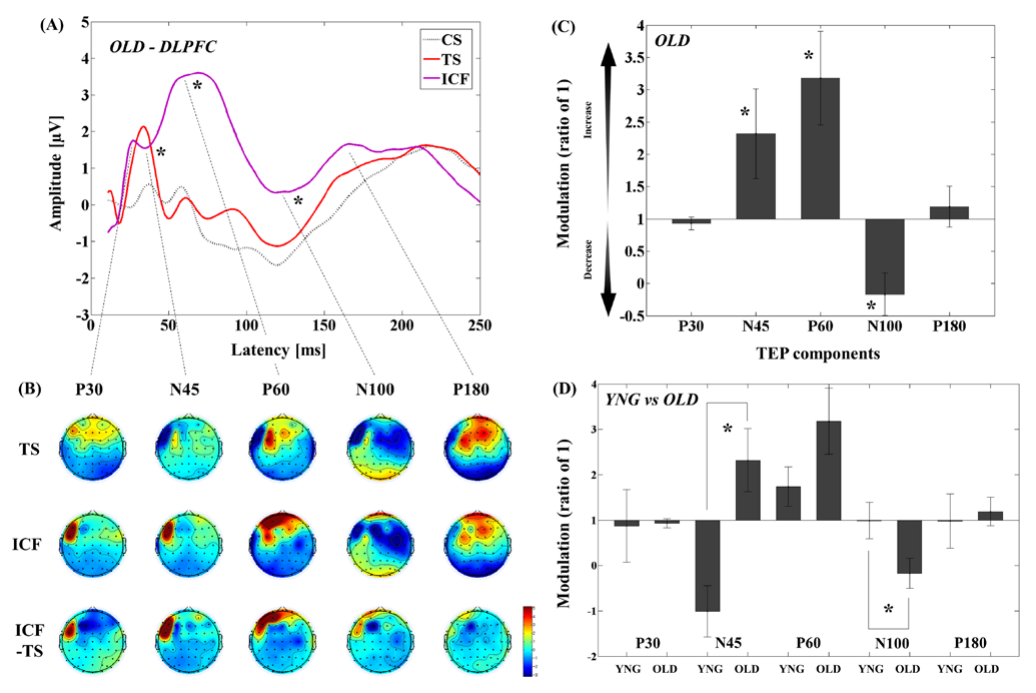
Modulation of TEPs by the DLPFC-ICF paradigm in older adults **(A)** The graph depicts TEP traces averaged across the older adults for subthreshold TMS (black dot line: CS), unconditioned TMS (red line: TS) and conditioned TMS (purple line: ICF; ISI = 10ms) at the left frontal ROI. **(B)** The illustration shows the EEG topographical plots for conditions of TS alone, ICF, and the difference between TS and ICF obtained from the DLPFC-ICF experiment. Each vertical column depicts the TEP topoplots for P30, N45, P60, N100, and P180 component from left to right. **(C)** The bar graph shows modulatory effects of the DLPFC-ICF on TEPs in the older adults. The ANOVA and post-hoc analyses revealed that there are significant modulations (p < 0.05) in N45, P60, and N100 TEPs with the DLPFC-ICF paradigm. **(D)** The bar graph showing cross-sectional comparisons between younger and older adults in the DLPFC–ICF paradigm. The older adults demonstrate a significant facilitation of amplitude on N45 TEP and a significant attenuation of amplitude on N100 TEP compared to the younger adults (p < 0.05).

The results of averaged TEP traces of older adults for CS alone, TS alone, and conditioned stimulus in ICF paradigm are shown in Figure 2A. A 3–way ANOVA and post-hoc analyses (α–level: 0.05) for TEP values of the ICF showed significant differences between TS and CS&TS on TEP N45 (t_11_ = -2.481, p = 0.031; TS < ICF; Cohen's *d* = 0.83, Power (1-β) = 0.745), P60 (t_11_ = -9.928, p < 0.0001; TS < ICF; Cohen's *d* = 3.01, Power (1-β) = 1.000), and N100 (t_11_ = -5.312, p < 0.0001; TS < ICF; Cohen's *d* = 1.65, Power (1-β) = 0.999) TEPs at the left frontal ROI (Supplementary Table S2). Figure 2B shows the EEG topographical plots for conditions of TS, ICF, and the difference between TS and ICF in the DLPFC-ICF experiment. The TEP amplitude modulations at the left frontal ROI are demonstrated in Figure 2C.

### Cross-sectional analyses of TEPs between younger and older adults by DLPFC-SICI and DLPFC-ICF

#### DLPFC-SICI differences between younger and older adults

Cross-sectional comparisons of TEP modulation between younger and older adults revealed that modulation of N100 TEP was significantly different (t_22_ = -2.975, p = 0.007; Cohen's *d* = 1.22, Power (1-β) = 0.815) between younger and older adults, with younger adults showing decrease while older adults showed increase in N100 TEP with SICI (Figure 1D and Supplementary Table S1; Supplementary Figure S2). In addition, there was no significant main effect of group (F_1,22_ = 0.467, p = 0.501) in the four-way ANOVA for the SICI paradigm, suggesting that the TEP difference in this paradigm was not come from the differences in single pulse TEP between the two groups.

#### DLPFC-ICF differences between younger and older adults

Cross-sectional comparisons of TEP modulations between younger and older adults demonstrated that the modulation of N45 TEP was significantly different (t_22_ = -3.721, p = 0.001; Cohen's *d* = 1.55, Power (1-β) = 0.952) between the two groups, with younger subjects demonstrating decrease while older subjects showed increase in N45 TEP. In contrast, the negative modulation of N100 TEP (t_22_ = 2.250, p = 0.035; Cohen's *d* = 0.92, Power (1-β) = 0.577) was significantly stronger in older adults compared to younger adults (Figure 2D and Supplementary Table S2; Supplementary Figure S2). Furthermore, the four-way ANOVA for the ICF paradigm showed no significant main effect of group (F_1,22_ = 0.465, p = 0.503), indicating that there was no direct effect of single pulse TEP differences on the observed TEP differences between the two groups in this paradigm.

### Correlation analyses

#### Correlations with age in DLPFC-SICI and DLPFC-ICF paradigms

**Figure 3:**
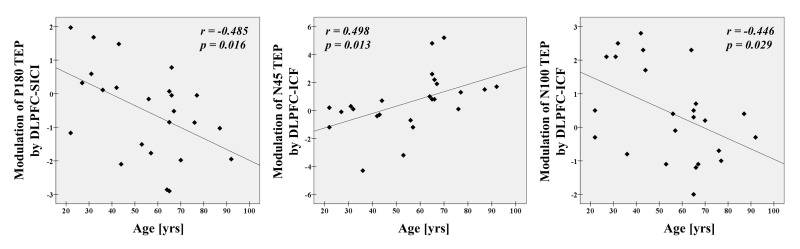
Age-related modulations on TEPs in all participants There are significant correlations between age and modulation of P180 TEP by DLPFC-SICI (r = -0.485, p = 0.016, N = 24), as well as between age and modulations of N45 (r = 0.498, p = 0.013, N = 24) and N100 TEPs by DLPFC-ICF (r = -0.446, p = 0.029, N = 24) at the left frontal ROI.

Correlation analyses in SICI paradigm for all participants revealed that age significantly correlated with modulation of P180 TEP (r = -0.485, p = 0.016, N = 24) at the left frontal ROI (Figure 3). In ICF paradigm, there were significant correlations with age in modulations of N45 (r = 0.498, p = 0.013, N = 24) and N100 (r = -0.446, p = 0.029, N = 24) TEPs at the left frontal ROI (Figure 3).

#### Correlations of TEP modulations within and between DLPFC-SICI and DLPFC-ICF

**Figure 4:**
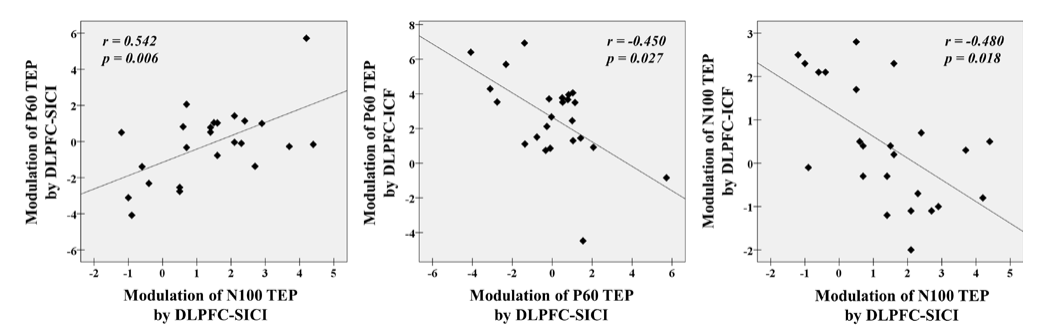
Neurophysiological relationship with SICI and ICF paradigms from the DLPFC There are significant correlations between modulations of P60 TEP and N100 TEP with DLPFC-SICI (r = 0.542, p = 0.006, N = 24), and further between a modulation of P60 TEP by DLPFC-SICI and a modulation of P60 TEP by DLPFC-ICF (r = -0.450, p = 0.027, N = 24) and between a modulation of N100 TEP by DLPFC-SICI and a modulation of N100 TEP by DLPFC-ICF (r = -0.480, p = 0.018, N = 24) at the left frontal ROI.

In SICI paradigm, there was a significant correlation between modulations of P60 and N100 TEPs (r = 0.542, p = 0.006, N = 24, Figure 4). For the ICF paradigm, there were no correlations between TEP modulations. Between SICI and ICF paradigms, we observed significant correlations of TEP modulations between P60 in SICI and P60 in ICF (r = -0.450, p = 0.027, N = 24), and between N100 in SICI and N100 in ICF (r = -0.480, p = 0.018, N = 24). Since N100 modulation by ICF was significantly associated with age, we did a partial correlation using age as a covariate and found that the positive correlation between modulations of N100 in SICI and ICF remained significant (r = -0.389, p = 0.033, N = 21). These correlations are depicted in Figure 4.

## DISCUSSION

### Summary of the study findings

This study revealed several important findings. First, in the SICI paradigm, older adults demonstrated less inhibition on N100 TEP compared to younger adults (Figure 1D). Second, ICF induced facilitation of N45 TEP among older adults, while N45 was inhibited in younger adults. Further, older adults showed less inhibition, with inhibition turned into facilitation, on N100 TEP compared to younger adults (Figure 2D). Third, we found that there was an excitatory and inhibitory (E/I) balance in terms of the modulations of P60 and N100 between the SICI and ICF paradigms that was maintained across age (Figure 4).

### Differences in SICI and ICF between younger and older adults

Several lines of evidence have shown that SICI in the motor cortex measured with MEP decreases with age [3, 12, 13]. However, age-related TEP changes in SICI in the DLPFC have not been previously studied. In the DLPFC, SICI induced facilitation of N100 among older adults, while younger adults had inhibition of N100, suggestive of a compensatory change in TEP due to a deterioration of GABA_A_ receptor-mediated inhibition in the older adults. The N100 component is thought to be generated mainly from GABA_B_ receptor-mediated inhibition [14-20]. In addition, at the same time, GABA_A_ receptor-mediated inhibition plays a crucial role in balancing the Up state (i.e., more depolarized) of membrane potentials in neural circuits, whereas GABA_B_ receptor-mediated inhibition contributes to the transformation from Up state into Down state (i.e., more hyperpolarized) of membrane potentials [21, 22]. Collectively, functional decline of GABA_A_ receptor-mediated inhibition during SICI with age may contribute to a membrane potential shift from Up state to Down state, which in turn results in an increase of N100 TEP amplitude in the SICI paradigm in older adults. Therefore, a compensatory mutual interaction with age between GABA_A_ and GABA_B_ receptor-mediated inhibitions may contribute to the increase of N100 TEP in the SICI paradigm.

In contrast, ICF induced a reduction in amplitude of N45 and N100 TEPs in older adults compared to younger adults. These results suggest balanced decreases of early phase of inhibitory N45, as well as late phase of inhibitory N100 TEPs due to a functional decline of glutamatergic receptor mediated excitation in older adults during ICF. The ICF indexes both NMDA receptor-mediated glutamertagic and GABA_A_ receptor-mediated activity [23-25], and the N45 component is thought to be associated with the function of GABA_A_ receptor-mediated inhibition [26]. Thus, a reduction in amplitude of N45 TEP with the DLPFC-ICF paradigm in older adults may be related to an aging-mediated functional decline of GABA_A_ergic inhibition as well as glutamertagic excitation. Of note, since all participants were not taking any psychotropic medications or recreational substances including tobacco, it is likely that there were no specific confounding factors that could have contributed to the observed differences between the two groups.

### Age-related modulations in SICI and ICF paradigms

Age was negatively correlated with a modulation of P180 TEP and positively correlated with a modulation of N45 TEP in the SICI paradigm. With ICF, age was negatively correlated with a modulation of N100 TEP. These results indicate that aging reduces the late phase of excitatory TEP (i.e., P180) and facilitates the early phase of inhibitory TEP (i.e., N45) as probed with SICI. In addition, aging seems to reduce the late phases of inhibitory TEP (i.e., N100) during ICF. The underlying physiology resulting in the P180 TEP is still not clear, but it is thought to represent excitatory activity. Potentially, the reduction of GABA_A_ receptor-mediated inhibitory function with age (i.e., reduction in SICI) could indirectly lower the counter balance of excitatory activity, resulting in the observed decrease of P180 TEP component. The N45 and N100 TEPs are thought to be associated with GABA_A_ receptor- and GABA_B_ receptor-mediated inhibitions respectively [18, 26]. Thus the TEP modulations observed with ICF paradigm relate to the decrease of GABA levels in the DLPFC that occurs with aging [1, 2].

### Neurophysiological relationship between SICI and ICF

We observed correlations between modulations of P60 and N100 TEPs induced by SICI, and further between P60 TEP modulations induced by SICI and ICF as well as between N100 TEP modulations induced by both paradigms. These results suggest an antagonistic or compensatory E/I relationship in TEP modulations within SICI paradigm itself as well as in TEP modulations between SICI and ICF. Furthermore, since no direct aging effects were observed on these relationships, these findings are likely to represent stable neurophysiological characteristics in healthy subjects.

First, in the SICI paradigm, the degree of facilitation of P60 TEP amplitude was positively correlated with the degree of facilitation of N100 TEP amplitude across all participants, which could be explained by an antagonistic E/I balance related to GABA_A_ receptor-mediated effects that occur irrespective of age (Figure 4: left). Second, between the ICF and SICI paradigms, the degree of facilitation of P60 by ICF was negatively correlated with the extent of inhibition of this component by SICI, which suggests a compensatory mechanism between glutamate receptor-mediated effects (i.e., P60 modulation) and GABA_A_ receptor-mediated effects (i.e., P60 modulation) in an age-independent way (Figure 4: middle). Third, the degree of inhibition of N100 by ICF was also negatively correlated with the extent of inhibition of N100 by SICI, which also suggests that there is a compensatory mechanism between glutamate receptor-mediated effects (i.e., N100 modulation) and GABA_A_ receptor-mediated effects (i.e., N100 modulation). (Figure 4: right).

These findings are consistent of the balance in the strength of glutamatergic and GABAergic neurotransmissions observed in rodent hippocampus and visual cortex [27, 28] and another recent human study [29]. The E/I balance is thought to be crucial to maintaining neural firing within a dynamic range [30], and failure to establish such a balance has been linked to several neurological and neuropsychiatric disorders [27, 31-33] such as schizophrenia [34, 35]. These recent results add further support to the notion of a balance in excitatory and inhibitory transmissions in human cortex, and suggest that TMS-EEG may be used to study aberrant E/I functioning non-invasively in prefrontal regions. Furthermore, the positive correlation between modulations of P60 and N100 TEPs within SICI paradigm is suggestive of a balanced E/I coupling between the two components during this paradigm.

There are some limitations to the current study. First, since we determined the site of stimulation over the DLPFC using a previously published method [43], the accuracy of the individual site of stimulation may not have been as precise compared to a magnetic resonance imaging-guided neuronavigation method. Nonetheless, the TEPs induced by SICI or ICF in the present study are consistent with those previously reported. Indeed, both methods with and without neuronavigation produce highly comparable results in previous studies [36, 37]. Second, as the present study has not included the neuroimaging modality such as MRS, future studies combining TMS-EEG with MRS would be very informative to validate the age-mediated relationship between the substance level of GABA/glutamate and TMS neurophysiology of SICI/ICF in the DLPFC. Third, the present study is limited by the lack of exploration of the functional significance of the neurophysiological findings. Future work should explore the relationship between these measures and formal neuropsychological tests of cognition. Lastly, although the sample size of subjects was determined based on previous TMS-EEG studies [11, 38], the sample size tested in the current study is relatively small. Thus, our findings warrant further investigation with larger sample size, leading to more precise and accurate TEP results.

In conclusion, we demonstrated age-related changes of TEP modulations induced by SICI and ICF paradigms in the DLPFC. Specifically, the present study showed that older adults had increased N100 TEP modulation by SICI, suggestive of an indirectly compensatory GABA_B_ receptor-mediated function through a deterioration of GABA_A_ receptor-mediated inhibition with age. Furthermore, ICF induced age-related changes in N45 and N100 TEP modulations, suggestive of a decline in GABA_A_ receptor-mediated inhibitory function with age. However, an E/I balance between glutamatergic and GABAergic functions indexed by ICF and SICI paradigms was maintained across age, suggesting a compensatory mechanism between the two. As such, the use of the SICI and ICF paradigms with TMS-EEG may hold potential for studying both of healthy and pathological forms of aging. For example, future studies may be able to assess whether an E/I balance in the prefrontal cortex plays a role in pathological forms of aging such as mild cognitive impairment and Alzheimer's disease.

## METHODS

### Participants

Twelve right-handed younger adults (6 female; aged 22–57 yrs; mean age 39 ± 12 yrs) and 12 right-handed older adults (6 female; aged 64–92 yrs; mean age 72 ± 9 yrs) participated in the present study. Younger adults between the ages of 18–59 and older adults over the age of 60, who met the following criteria were eligible to participate in this study: (i) no history of neurological disorders including seizure or stroke, (ii) no history of neuropsychiatric disorders, (iii) normal cognitive function, (iv) no history of alcohol or other drug abuse/dependence, and (v) did not smoke, use recreational substances or psychiatric prescription medications. All participants were screened with the Structured Clinical Interview for DSM–IV Axis I Disorders prior to the study. The experiment was conducted in accordance with the Declaration of Helsinki and approved by the Research Ethics Board at the Centre for Addiction and Mental Health.

### TMS procedure and electromyography measure

Monophasic TMS pulses were administered to the left M1 using a 70 mm figure-of-eight coil, and two Magstim 200 stimulators (Magstim Company Ltd., UK) connected via a Bistim module. MEP data were collected using commercially available software, Signal (Cambridge Electronics, UK). During testing, the participants sat in a chair and were instructed to keep their eyes open and relax throughout the study. First, the hot spot for the right first dorsal interosseous muscle to evoke the largest MEP over M1 was determined. Then, the individual intensity of RMT to induce more than 50μV MEP amplitude of the same muscle five times out of 10 was determined. Finally, we determined the individual intensity to induce 1mV peak-to-peak MEP amplitude of the same muscle.

### SICI and ICF measures

SICI is thought to be mediated by GABA_A_ receptors [3-10], which is indexed at (2-4 ms) interstimulus intervals (ISI) between the first subthreshold (e.g., 80% of RMT) and second suprathreshold pulse (e.g., suprathreshold intensity to induce 1mV peak to peak MEP amplitude). Further, it is thought that SICI consists of at least two phases of inhibition: the conditioning pulse elicits short-lasting inhibitory postsynaptic potentials in corticospinal neurons through activation of a low-threshold cortical inhibitory circuit mediated by the GABA_A_ receptors, which inhibits action potential generation in these neurons by a suprathreshold second pulse [3, 9, 11-14]. In addition, SICI is maximal when the suprathreshold second TMS pulse is administered at the intensity to induce 1mV peak-to-peak MEP amplitudes [9, 10, 15, 16]. This implies that SICI is a net inhibition consisting of low-threshold inhibitory and higher-threshold facilitatory effects of the conditioning pulse on the test MEP [9, 17].

In contrast to SICI, the ICF paradigm leads to a facilitated MEP when the subthreshold conditioning pulse is delivered 7-20 ms before a suprathreshold test pulse [3, 5, 18]. The range of such ISIs for ICF may reflect slow excitatory postsynaptic potentials mediated by NMDA receptor activation [19]. Compared to SICI, the physiological mechanism of ICF is less clear [20], however, it is thought that ICF measures an excitatory cortical neural circuit that is dissociable from the SICI network [5]. ICF is assumed to be a net facilitation consisting of prevailing facilitation and weaker inhibition that comes from the tail of the GABA_A_ receptor-mediated inhibitory postsynaptic potentials as a component of SICI, which lasts for approximately 20 ms [11, 21]. We determined the site of stimulation over the DLPFC using an EEG-cap (electrode F5) approximation method [43].

SICI and ICF were examined according to the established methods [24, 39, 40]. For SICI, the ISI of 2 ms was used to avoid contamination by SICF [41] and for ICF an ISI of 10 ms was applied. The CS intensity was set at 80% of RMT and TS intensity was set to evoke 1mV peak-to-peak MEP amplitude when delivered alone.

### EEG recording and pre-processing

EEG was acquired through a 10–10 montage of 64-channel Neuroscan Synamps 2 EEG system with a TMS-compatible EEG cap (Compumedics Neuroscan, Australia). All electrodes were referenced to an electrode positioned posterior to Cz. Recording electrodes impedance was kept to ≤5kΩ during the experiment. EEG signals were recorded at direct current with a sampling rate of 20 kHz and an online lowpass filter of 200 Hz was applied. EEG data were processed offline using MATLAB (R2014a, The MathWorks, MA, USA) and EEGLAB toolbox [42]. All data were down–sampled to 1000 Hz for analyses.

### EEG signal processing

The continuous EEG data were epoched from -1000 ms to 2000 ms relative to the TMS pulse. Baseline correction was conducted with respect to the pre-stimulus interval -500 ms to -110 ms. EEG data was re-segmented from 10 ms to 2000 ms post-TMS to remove the TMS artifact. EEG data were visually inspected to eliminate trials and channels that were highly contaminated with noise. More than 80% of trials and 95% of channels survived artifact rejection. Independent component analysis (ICA) was subsequently applied to minimize and remove typical TMS-related decay artifacts that appear at very short early latency immediately after the TMS pulse and eye-related artifacts and remaining muscle activity related components. Following ICA, the Butterworth, zero-phase shift 1-55 Hz band pass filter (24dB/Oct) and notch filter were applied. In each subject, the number of ICA components that were removed from original 62 ICA components was not greater than 20%. Finally, data was re-referenced to the average electrode for analyses of the TEP.

### TEP data analyses in SICI and ICF paradigms

For TEP analyses, the effect of SICI or ICF paradigm on the individual amplitude of TEP components (P30, N45, P60, N100 and P180) was computed at each condition (i.e., CS, TS, and CS&TS: SICI or ICF) at the 5 ROIs (Supplementary Figure S1) obtained from the DLPFC–SICI and DLPFC–ICF experiments. Further, based on our prior analytic method, we did not subtract the CS TEP from paired pulse TEP [11]. In the TEP analyses, we calculated the modulation of TEPs by the ratio of CS&TS / TS. In addition, in negative polarity of TEP components (i.e., N45 and N100), an increase in the amplitude means that the trough of TEP becomes deeper (i.e., more negative), and vice versa, whereas, in positive polarity of TEP components (i.e., P30, P60, P180), an increase in the amplitude means that peak of TEP becomes higher (i.e., more positive), and vice versa.

### Statistical analyses

SPSS version 19.0 was used for statistical analysis. The following assessments were performed: 1) SICI or ICF effects on amplitude of TEP components, 2) group differences of amplitude of TEP components between younger and older adults, and 3) correlation analyses between amplitude changes of TEP component and age for each SICI or ICF paradigm as well as between amplitude changes of TEP component in both SICI and ICF paradigms.

ANOVA with Bonferroni correction (i.e., α = 0.05/2) were applied to amplitudes of TEP components to examine the significant effects of SICI or ICF on TEPs with factors described below, separately. We performed a three-way ANOVA with ROIs (i.e., 5 levels: 5 ROIs, see Supplementary Figure S1), TEP components (i.e., 5 levels: P30, N45, P60, N100, and P180), and conditions (i.e., 2 levels: TS and CS&TS) as within-subject factors for both DLPFC-SICI and DLPFC-ICF paradigms, separately. Cross-sectional comparison analyses were performed using post–hoc t–tests, focusing on the significant results demonstrated in the Bonferroni corrected ANOVA above (α–level: 0.025). In addition, to confirm the effect of single pulse TEP differences between younger and older groups, we performed a four-way ANOVA for TEP data of all participants that incorporated “group” as a between-subject factor into the above three-way ANOVA model in both paradigms, separately.

The correlation analyses between age and TEP modulations with SICI as well as ICF paradigms at the left frontal ROI were performed with Pearson's correlation coefficient for the significant results obtained from the above ANOVA. We further explored correlations between TEP modulations with SICI and ICF paradigms. A significant level of α = 0.05 was applied. The sample size of subjects was based on a previous study in 12 younger healthy controls [11].

## SUPPLEMENTAL MATERIAL FIGURES AND TABLES



## Abbreviations

(ANOVA): Analyses of variance
(CS): conditioning stimulus
(DLPFC): dorsolateral prefrontal cortex
(EEG): electroencephalography
(E/I): excitatory and inhibitory
(GABA): gamma-aminobutyric acid
(GAD): glutamic acid decarboxylase
(ICA): independent component analysis
(ISI): interstimulus intervals
(ICF): intracortical facilitation
(MRS): magnetic resonance spectroscopy
(MEP): motor evoked potential
(M1): primary motor cortex
(NMDA): N-methyl-D-aspartate
(ROI): region of interest
(RMT): resting motor threshold
(SICI): short interval intracortical inhibition
(TS): test stimulus
(TMS): transcranial magnetic stimulation
(TEP): TMS-evoked potential
