# Supplementary material for: Characterization of the influence of age on GABAA and glutamatergic mediated functions in the dorsolateral prefrontal cortex using paired-pulse TMS-EEG
Source: Aging (Albany NY). 2017 Feb 16;9(2):556–67. doi: 10.18632/aging.101178 (PMC5361681; doi:10.18632/aging.101178)
Supplement: Supplementary file 1 [file aging-09-556-s001.pdf]

SUPPLEMENTARY MATERIAL

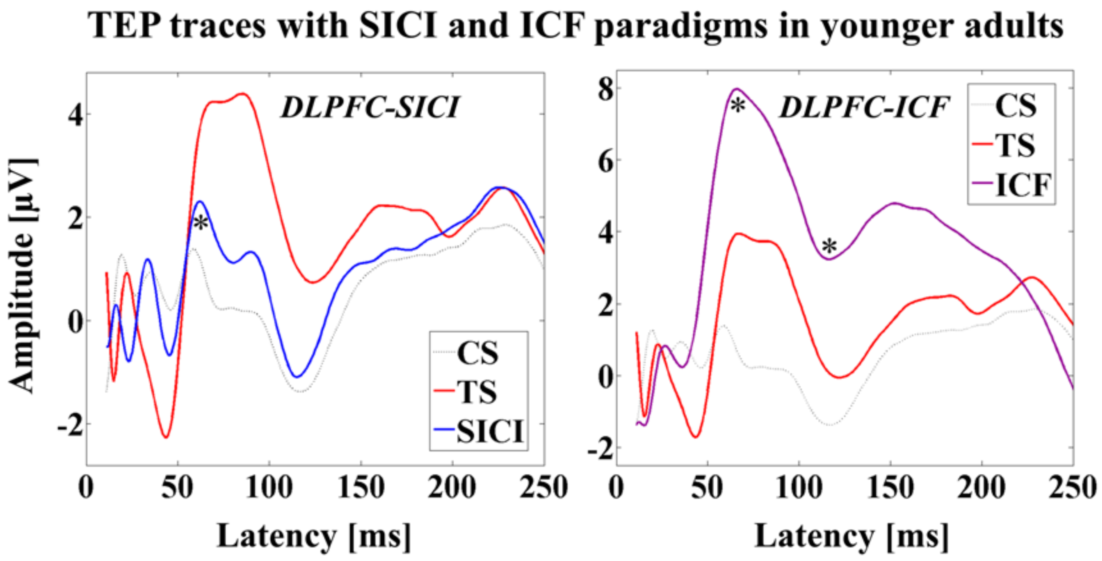

Supplementary Figure S1. The selection of electrodes comprising each ROI. Left frontal ROI, right frontal ROI, midline central ROI, left central ROI, and right central ROI represent the left DLPFC, right DLPFC, midline central, left M1, right M1 areas, respectively.

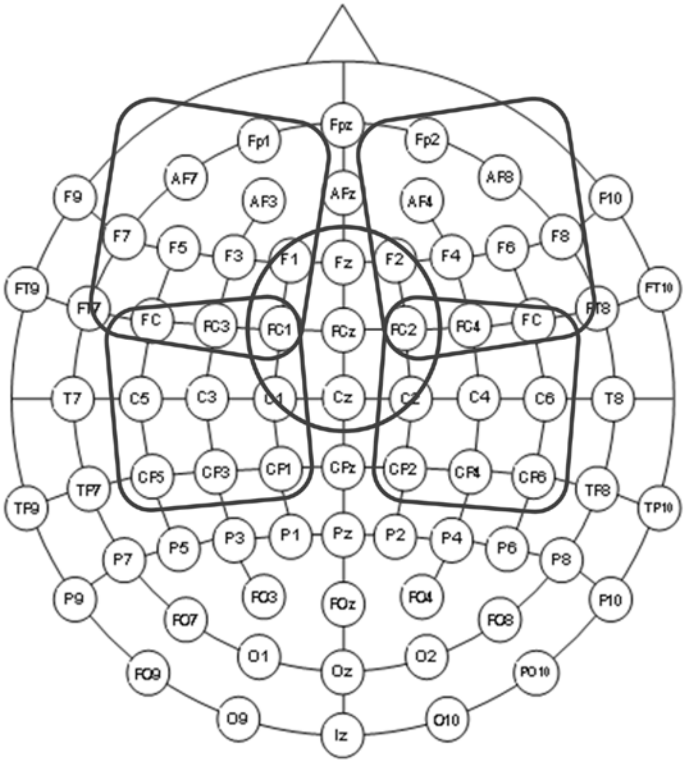

Supplementary Figure S2. TEP traces with SICI and ICF paradigms at the left DLPFC in younger adults. P60 TEP was significantly attenuated by the SICI paradigm, while it was significantly increased by the ICF paradigm. Further, N100 TEP was significantly attenuated by the ICF paradigm. These results are derived from our previously published data [11].

Supplementary Table S1. The significant results of 3-way ANOVA for the TEP amplitudes in DLPFC-SICI.

3-way ANOVA (α = 0.025)			
**Main effects**		η^2	**Observed power**
ROI	$F_{4,44} = 23.561, p < 0.0001$	0.682	1.000
TEP component	$F_{4,44} = 63.147, p < 0.0001$	0.852	1.000
Condition	$F_{1,11} = 7.396, p = 0.020$	0.402	0.698
**Interactions**		η^2	**Observed power**
ROI–by–TEP component	$F_{16,176} = 24.673, p < 0.0001$	0.692	1.000
ROI–by–Condition	$F_{4,44} = 4.580, p = 0.004$	0.294	0.920
ROI–by–TEP component–by–Condition	$F_{16,176} = 1.956, p = 0.018$	0.151	0.955
**MANOVA (α = 0.05)**			
**Simple main effects**		η^2	**Observed power**
ROI	$F_{4,8} = 15.200, p = 0.001$	0.884	0.998
TEP component	$F_{4,8} = 75.604, p < 0.0001$	0.974	1.000
Condition	$F_{1,11} = 7.396, p = 0.02$	0.402	0.698
**Simple interactions**		η^2	**Observed power**
ROI–by–TEP component; TEP P60	$F_{4,8} = 9.609, p = 0.004$	0.828	0.970
ROI–by–TEP component; TEP N100	$F_{4,8} = 13.077, p = 0.001$	0.881	0.998
ROI–by–TEP component; TEP P180	$F_{4,8} = 78.424, p < 0.0001$	0.975	1.000
ROI–by–TEP component; the left frontal ROI	$F_{4,8} = 114.567, p < 0.0001$	0.983	1.000
ROI–by–Condition; TS	$F_{4,8} = 14.234, p = 0.001$	0.877	0.997
ROI–by–Condition; CS.TS (SICI)	$F_{4,8} = 6.563, p = 0.012$	0.766	0.880
ROI–by–Condition; the left frontal ROI	$F_{1,11} = 26.035, p < 0.0001$	0.703	0.996
TEP component–by–Condition; TS	$F_{4,8} = 54.277, p < 0.0001$	0.964	1.000
TEP component–by–Condition; CS.TS (SICI)	$F_{4,8} = 51.501, p < 0.0001$	0.963	1.000
ROI–by–TEP component–by–Condition; TEP P60–by–TS	$F_{4,8} = 11.703, p = 0.002$	0.854	0.989
ROI–by–TEP component–by–Condition; TEP P60–by–CS.TS (SICI)	$F_{4,8} = 5.224, p = 0.023$	0.723	0.791
ROI–by–TEP component–by–Condition; TEP N100–by–TS	$F_{4,8} = 4.183, p = 0.041$	0.671	0.675
ROI–by–TEP component–by–Condition; TEP N100–by–CS.TS (SICI)	$F_{4,8} = 29.325, p < 0.0001$	0.936	1.000
ROI–by–TEP component–by–Condition; TEP P180–by–TS	$F_{4,8} = 27.792, p < 0.0001$	0.933	1.000
ROI–by–TEP component–by–Condition; TEP P180–by–CS.TS (SICI)	$F_{4,8} = 22.532, p < 0.0001$	0.918	1.000
ROI–by–TEP component–by–Condition; the left frontal ROI–by–TS	$F_{4,8} = 44.765, p < 0.0001$	0.957	1.000
ROI–by–TEP component–by–Condition; the left frontal ROI–by–CS.TS (SICI)	$F_{4,8} = 70.51, p < 0.0001$	0.972	1.000
ROI–by–TEP component–by–Condition; the left frontal ROI–by–TEP P60	$F_{1,11} = 25.195, p < 0.0001$	0.696	0.995
ROI–by–TEP component–by–Condition; the left frontal ROI–by–TEP N100	$F_{1,11} = 19.311, p = 0.001$	0.637	0.979
ROI–by–TEP component–by–Condition; the left frontal ROI–by–TEP P180	$F_{1,11} = 8.905, p = 0.012$	0.447	0.775
**Post-hoc paired t-test (α = 0.05)**		***d***	**Power (1-β)**
TS > CS.TS (SICI); TEP P60 at the left frontal ROI	$t_{11} = 5.019, p < 0.0001$	1.19	0.963
TS > CS.TS (SICI); TEP N100 at the left frontal ROI	$t_{11} = 4.394, p = 0.001$	1.54	0.998

TS > CS.TS (SICI); TEP P180 at the left frontal ROI	t_{11} = 2.984, p = 0.012	0.69	0.587

*MANOVA: multivariate analysis of variance

The significant results of comparison analysis between young and old participants in the modulation of TEP by DLPFC–SICI

Post-hoc independent t-test (α = 0.05)		*d*	Power (1-β)
YNG < OLD participants (ratio of 1); TEP N100 modulation at the left frontal ROI	t_{22} = -2.975, p = 0.007	1.22	0.815

Supplementary Table S2. The significant results of 3-way ANOVA for the TEP amplitudes in DLPFC-ICF.

3-way ANOVA (α = 0.025)			
**Main effects**		η^2	**Observed power**
ROI	$F_{4,44}$ = 43.393, p < 0.0001	0.798	1.000
TEP component	$F_{4,44}$ = 76.432, p < 0.0001	0.874	1.000
Condition	$F_{1,11}$ = 50.577, p < 0.0001	0.821	1.000
**Interactions**		η^2	**Observed power**
ROI–by–TEP component	$F_{16,176}$ = 21.770, p < 0.0001	0.664	1.000
ROI–by–Condition	$F_{4,44}$ = 10.224, p < 0.0001	0.541	1.000
TEP component–by–Condition	$F_{4,44}$ = 4.752, p = 0.003	0.302	0.930
ROI–by–TEP component–by–Condition	$F_{16,176}$ = 6.272, p < 0.0001	0.363	1.000
**MANOVA (α = 0.05)**			
**Simple main effects**		η^2	**Observed power**
ROI	$F_{4,8}$ = 24.600, p < 0.0001	0.925	1.000
TEP component	$F_{4,8}$ = 42.624, p < 0.0001	0.955	1.000
Condition	$F_{1,11}$ = 50.577, p < 0.0001	0.821	1.000
**Simple interactions**		η^2	**Observed power**
ROI–by–TEP component; TEP N45	$F_{4,8}$ = 7.613, p = 0.008	0.792	0.924
ROI–by–TEP component; TEP P60	$F_{4,8}$ = 27.033, p < 0.0001	0.931	1.000
ROI–by–TEP component; TEP N100	$F_{4,8}$ = 8.483, p = 0.006	0.809	0.949
ROI–by–TEP component; the left frontal ROI	$F_{4,8}$ = 34.372, p < 0.0001	0.945	1.000
ROI–by–Condition; TS	$F_{4,8}$ = 9.079, p = 0.005	0.819	0.962
ROI–by–Condition; CS.TS (ICF)	$F_{4,8}$ = 15.264, p = 0.001	0.884	0.998
ROI–by–Condition; the left frontal ROI	$F_{1,11}$ = 36.439, p < 0.0001	0.768	1.000
TEP component–by–Condition; TS	$F_{4,8}$ = 44.473, p < 0.0001	0.957	1.000
TEP component–by–Condition; CS.TS (ICF)	$F_{4,8}$ = 36.340, p < 0.0001	0.948	1.000
TEP component–by–Condition; TEP N45	$F_{1,11}$ = 9.462, p = 0.011	0.462	0.799
TEP component–by–Condition; TEP P60	$F_{1,11}$ = 29.791, p < 0.0001	0.730	0.999
TEP component–by–Condition; TEP N100	$F_{1,11}$ = 13.587, p = 0.004	0.553	0.918

ROI–by–TEP component–by–Condition; TEP N45–by–TS	$F_{4,8} = 20.221, p < 0.0001$	0.910	1.000
ROI–by–TEP component–by–Condition; TEP N45–by–CS.TS (ICF)	$F_{4,8} = 4.281, p = 0.038$	0.682	0.699
ROI–by–TEP component–by–Condition; TEP P60–by–TS	$F_{4,8} = 17.688, p < 0.0001$	0.898	1.000
ROI–by–TEP component–by–Condition; TEP P60–by–CS.TS (ICF)	$F_{4,8} = 65.866, p < 0.0001$	0.971	1.000
ROI–by–TEP component–by–Condition; TEP N100–by–TS	$F_{4,8} = 12.109, p = 0.002$	0.858	0.991
ROI–by–TEP component–by–Condition; TEP N100–by–CS.TS (ICF)	$F_{4,8} = 8.913, p = 0.005$	0.817	0.959
ROI–by–TEP component–by–Condition; the left frontal ROI–by–TS	$F_{4,8} = 42.030, p < 0.0001$	0.955	1.000
ROI–by–TEP component–by–Condition; the left frontal ROI–by–CS.TS (ICF)	$F_{4,8} = 13.158, p = 0.001$	0.868	0.995
ROI–by–TEP component–by–Condition; the left frontal ROI–by–TEP N45	$F_{1,11} = 6.156, p = 0.031$	0.359	0.619
ROI–by–TEP component–by–Condition; the left frontal ROI–by–TEP P60	$F_{1,11} = 98.561, p < 0.0001$	0.900	1.000
ROI–by–TEP component–by–Condition; the left frontal ROI–by–TEP N100	$F_{1,11} = 28.217, p < 0.0001$	0.720	0.998
**Post-hoc paired t-test (α = 0.05)**		***d***	**Power (1-β)**
TS < CS.TS (ICF); TEP N45 at the left frontal ROI	$t_{11} = -2.481, p = 0.031$	0.83	0.745
TS < CS.TS (ICF); TEP P60 at the left frontal ROI	$t_{11} = -9.928, p < 0.0001$	3.01	1.000
TS < CS.TS (ICF); TEP N100 at the left frontal ROI	$t_{11} = -5.312, p < 0.0001$	1.65	0.999

*MANOVA: multivariate analysis of variance

The significant results of comparison analysis between young and old participants in the modulation of TEP by DLPFC-ICF

Post-hoc independent t-test (α = 0.05)		*d*	Power (1-β)
YNG < OLD participants (ratio of 1); TEP N45 modulation at the left frontal ROI	$t_{22} = -3.721, p = 0.001$	1.55	0.952
YNG > OLD participants (ratio of 1); TEP N100 modulation at the left frontal ROI	$t_{22} = 2.250, p = 0.035$	0.92	0.577
